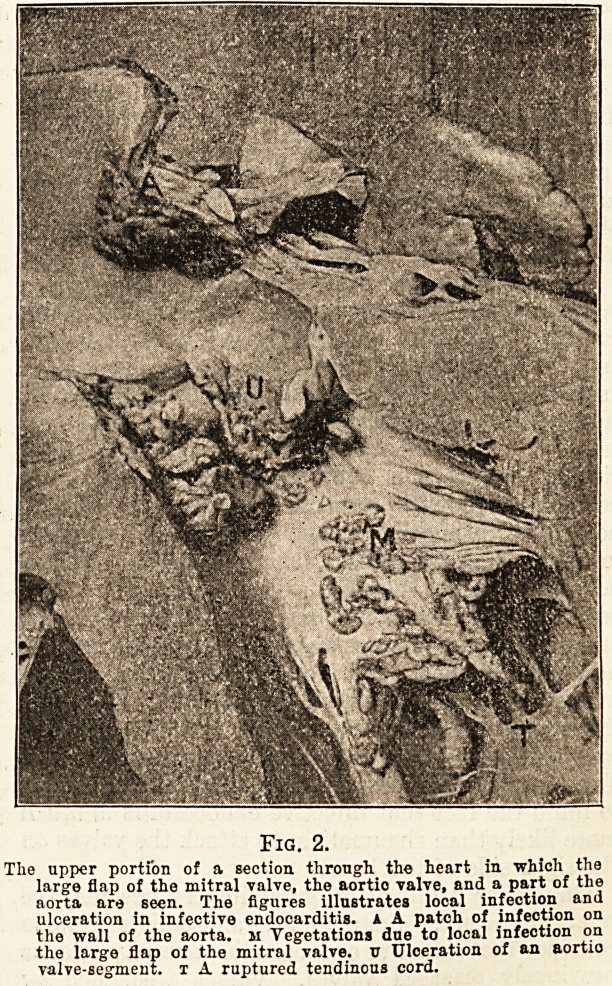# Infective Endocarditis

**Published:** 1908-07-18

**Authors:** Theodore Fisher


### INFECTIVE ENDOCARDITIS.

By THEODORE FISHER, M.D., F.R.C.P.

The term infective might perhaps be correctly applied to any form of endocarditis. There is no doubt that rheumatic endocarditis deserves the name infective, and even calcareous disease of the aortic valve may possibly be due to infection with micro-organisms. The word infective, however, is generally applied to those forms of disease of the cardiac valves set up by micro-organisms which are capable of giving rise to the formation of actual pus either within the heart or in other parts of the body.

Infective endocarditis has also in times past been called ulcerative because ulceration of the valves is frequently present, and the alternative term malignant has been applied to the disease because it nearly always proves fatal to the person attacked. Ulceration, though frequent, is not by any means an invariable feature. A much more distinctive mark of this variety of disease of the valves is the large size of the vegetations. While in rheumatism and chorea the vegetations are minute, being rarely larger than small grains of sand, in infective endocarditis they may reach the size of a small marble. (See fig. 1.)

They need not, however, be nearly so large as in this specimen in order to afford evidence by their size that infective endocarditis is present. In some cases of death from such diseases as suppurative nephritis, suppuration within the bile ducts, or infective

Fig. 1.

A large vegetation on an aortic valve-segment in infective endocarditis. The channel of the aorta lies above and the upper portion of the cavity of the left ventricle below. The vegetation is sufficiently large almost to block the outlet from the ventricle.

osteomyelitis, the vegetations may be seen in comparatively early stages, but even then they are generally much larger than those seen after rheumatism or chorea. In passing, a case of somewhat different character may be mentioned in which the presence of moderately large vegetations was of interest. A boy, aged 18—apparently not obviously ill at the time—died in the course of a few hours from urgent dyspnœa. At the autopsy fluid poured from the trachea and bronchi on section of the lungs, while an unexpected and sug-

Fig. 2.

The upper portion of a section through the heart in which the large flap of the mitral valve, the aortic valve, and a part of the aorta are seen. The figures illustrates local infection and ulceration in infective endocarditis. A A patch of infection on the wall of the aorta. M Vegetations due to local infection on the large flap of the mitral valve. U Ulceration of an aortic valve-segment. T A ruptured tendinous cord.

gestive feature was the presence of vegetations on the cusps of the aortic valve, obviously of infective nature. Œdema of the lungs is an obscure disease, but the case seemed to show that it may arise as a complication of a blood infection, the evidence of which was recently formed, yet comparatively large, vegetations on the aortic valve.

To return, however, to the appearances of infective endocarditis, while the large size of the vegetations may be the only indication that the disease is not of comparatively simple nature, other more striking features may be present. Ulceration of valve-segments has already been mentioned. Another appearance which perhaps more than any other justifies the name "infective," is the evidence of local infection, the most common result of which is a roughened surface and the presence of vegetations where the blood-stream impinges after it has passed over the diseased valve. In fig. 2 this feature of infective endocarditis is well illustrated. The cusps of the aortic valve are extensively diseased, and above them a large fibrinous patch indicates that the infected blood stream has attacked the aorta. Below the aortic valve also vegetations mark the course of a regurgitant blood stream over the ventricular surface of the large flap of the mitral valve. This specimen also shows ulcerative changes. Close to the letter U it will be noticed that there is ulceration of the aortic valve-segments and near the letter T is the broken end of one of the chordæ tendineæ which has given way as the result of ulceration. This specimen shows, therefore, two important, but not invariably present, features of infective endocarditis—local infection and ulceration. Evidence of much more severe local infection may, however, be present. I have seen an abscess in the interventricular septum, and an aneurysm of the aorta due to destruction of all the coats down to the sheath of the vessel. A hole through the centre of a valve is also not uncommon.

Brief reference may be made to the designation malignant, sometimes applied to infective endocarditis. Infective endocarditis is, without doubt, nearly always fatal, but cases of recovery have been recorded, from apparent examples of this disease, under the use of antistreptococcic serum. Whether, however, all such cases have or have not been rightly diagnosed is another matter. Yet in fatal cases of infective endocarditis there are not uncommonly evidences in the post-mortem room of local healing. I have seen, for example, the ulcerated edge of a more than half destroyed aortic valve-segment healed over, and extensive puckering in the cusps of the tricuspid valve where active disease was no longer present. I have also recorded a case in which a granular calcareous mass in the large flap of the mitral valve was apparently the sequel of infective endocarditis which had completely healed. The mention of disease in the tricuspid valve brings to mind the fact that infective endocarditis is much more likely than rheumatism to attack the valves on the right side of the heart.

One other detail of no great importance may be referred to here. Some morbid anatomists seem to consider that infective endocarditis generally attacks previously diseased valves. Valves weakened by previous inflammation one would expect to be more vulnerable to the attacks of microbes; yet, although this is, no doubt, true, the thickening of segments of a valve seen in infective endocarditis is undoubtedly in very many instances not due to an antecedent attack of endocarditis of simple nature. In this connection it may be well to mention that infective endocarditis is by no means always an acute disease. It may sometimes last for several months or even over a year. In these long-standing cases the presence of fibroid thickening of the valves is easily explained.

To return to fig. 2, the fibrinous granular-looking masses which form the vegetations readily suggest the liability to embolism, which is so common in this disease. The most important organ to suffer is the brain, and the emboli there may be multiple. I have seen a case where hemiplegia had been produced by embolus of one middle cerebral artery, and several weeks later a similar embolus, which proved fatal, became impacted in the corresponding artery on the opposite side. Sometimes, but by no means always, the embolus is infective. It may produce softening of the wall of an artery in which it lodges; with aneurysm as a result. Should an aneurysm of a cerebral artery burst, it is needless to say that cerebral hæmorrhage is the result. This may occur in very early life. It may be added that during childhood a septic embolus is virtually the only cause of cerebral hæmorrhage, though it is scarcely necessary to remark, not of hemiplegia. One other result of the infective character of an embolus is suppuration. Suppuration as the result of emboli may occur in various parts of the body, in the brain, in the spleen, in the kidneys, or under the skin, and less frequently in the liver. This scattered suppuration at one time gave rise to another name for the disease —arterial pyæmia. It may be repeated, however, that emboli in cases of infective endocarditis need manifest no qualities which suggest that they have been infective; that is to say, neither softening of arteries nor suppuration need follow their presence. As in other cases of cardiac disease, they may lead only to the formation of simple infarcts.

Here it may be mentioned that sometimes a clinical symptom present in infective endocarditis is assigned incorrectly to an infarct. This symptom is hæmaturia. In my student days I noted this error, and it has very frequently been brought home to me since. The cause of the hæmaturia is nephritis. In infective endocarditis an acute interstitial nephritis is not uncommon, which may occasionally lead to the formation of large white kidneys of considerable size. Large white kidneys, it is worthy of note, produced by interstitial changes, do not occasion general subcutaneous œdema, and consequently nephritis may not be suspected. It may be added that infarcts in the kidney, if they lead to hæmaturia, occasion hæmorrhage of very trivial and passing character.

In closing these few remarks upon infective endocarditis, the source of infection deserves comment. As indicated earlier, in many suppurative diseases early evidences of infection of the cardiac valves may be seen. Again, in death from more chronic diseases such as chronic gastric ulcer, carcinoma of the stomach, and tuberculosis of the lungs, infective endocarditis may be present which has obviously existed for several weeks or months. I have met with well-marked infective endocarditis in all these diseases. In the majority of cases, however, no source of infection can be discovered.

## Figures and Tables

**Fig. 1. f1:**
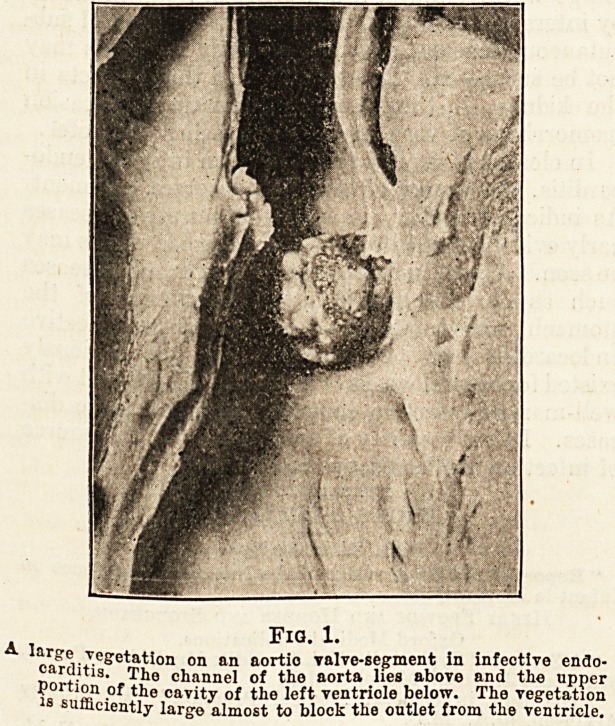


**Fig. 2. f2:**